# Real-Time Ultrasound Elastography in Multiple Sclerosis Spasticity: Comparison with Clinical and Neurophysiological Measures

**DOI:** 10.3390/jcm15114095

**Published:** 2026-05-26

**Authors:** Eleni Bakola, Marianna Papadopoulou, Maria-Ioanna Stefanou, Athanasios K. Chasiotis, Stella Fanouraki, Angeliki-Erato Sterpi, Dimitrios Kitsos, Georgios Tsivgoulis

**Affiliations:** 1 Second Department of Neurology, National and Kapodistrian University of Athens, School of Medicine, Attikon University Hospital, 12462 Athens, Greece; elbakola@yahoo.gr (E.B.); marianna421@hotmail.co.uk (M.-I.S.); thanosch1@gmail.com (A.K.C.); stelfanou@gmail.com (S.F.); angste1995@gmail.com (A.-E.S.); dkitsos@icloud.com (D.K.); tsivgoulisgiorg@yahoo.gr (G.T.); 2 Department of Physiotherapy, University of West Attica, 12243 Athens, Greece

**Keywords:** multiple sclerosis, spasticity, real-time elastography, muscle stiffness, H-reflex

## Abstract

**Background**: Spasticity is a common and disabling symptom of multiple sclerosis (MS), yet its assessment remains challenging. Clinical scales such as the Ashworth Scale (AS) evaluate resistance to passive movement, whereas neurophysiological measures (e.g., H-reflex, F-wave) provide objective indices of α-motoneuron excitability but correlate inconsistently with clinical severity. Real-time ultrasound elastography (RTE) enables semi-quantitative, in vivo assessment of muscle stiffness, while the recently introduced Muscle Elastography Multiple Sclerosis Score (MEMSs) aims to classify muscle spasticity in MS. **Objective**: To evaluate the utility of RTE using MEMSs for the objective assessment of muscle status in MS patients with spasticity, and to compare elastographic findings with clinical and neurophysiological measures. **Methods**: In this single-center study, 26 MS patients (diagnosed according to the 2017 McDonald criteria) and age- and sex-matched healthy controls (*n* = 27) were enrolled. Spasticity was graded using the AS. All participants underwent bilateral RTE of the gastrocnemius muscle, with images independently scored by two blinded neurologists using the 5-point MEMSs scale. Neurophysiological assessment included the soleus H-reflex, with calculation of H/M ratios. Correlations were analyzed using Pearson’s coefficient, and inter-observer reliability was assessed. **Results**: No consistent or statistically robust associations were found between clinical spasticity severity (AS scores) and either neurophysiological or elastographic parameters. Several MEMSs did not correlate reliably with AS grades, and neurophysiological measures showed limited discriminatory ability between MS patients and healthy controls. Correlations between neurophysiological and elastography parameters were weak to moderate (ρ ranging from −0.49 to 0.45). Inter-observer reliability of MEMSs scoring ranged from poor to moderate across the examined muscle groups, with Cohen’s κ values ranging from −0.02 to 0.54. **Conclusions**: RTE using MEMSs did not demonstrate sufficient validity or reproducibility for assessing muscle spasticity in MS, showing poor agreement with both clinical and neurophysiological measures.

## 1. Introduction

Ultrasound elastography is an imaging technique that evaluates tissue stiffness by measuring deformation in response to an external force, based on the principle that stiffer tissues deform less than softer ones. First introduced by Ophir and colleagues in 1991 [1], early elastographic methods used external compression and ultrasound-based displacement tracking to estimate tissue strain and generate qualitative maps of tissue elasticity, known as elastograms. This approach, later termed compression elastography, evolved into commercially available strain imaging systems in the early 2000s, providing real-time, color-coded visualization of tissue stiffness superimposed on conventional B-mode ultrasound, and finding widespread clinical application in lesion characterization [2].

In recent decades, real-time elastography (RTE) has gained broad clinical acceptance, particularly in oncologic imaging of liver, breast, and prostate lesions [3,4]. It is also applied in thyroid, gynecologic, and musculoskeletal imaging [5,6]. Elastograms are displayed as color-coded maps superimposed on B-mode ultrasound, illustrating tissue stiffness from soft to hard in a semi-quantitative manner, as applied in the assessment of breast lesions [3].

Spasticity, defined as a velocity-dependent increase in muscle tone due to hyperexcitability of the stretch reflex following upper motor neuron lesions, is a common and often distressing symptom in multiple sclerosis (MS) affecting over 80% of patients over the course of the disease and should be distinguished from rigidity, a velocity-independent increase in resistance to passive movement seen in extrapyramidal disorders such as Parkinson’s disease [7]. In MS, spasticity results from demyelination and axonal damage within central motor pathways, leading to loss of inhibitory supraspinal control over spinal reflex circuits [8]. It often coexists with motor weakness and disability, and its severity may or may not directly correlate with the degree of motor deficit [9]. It contributes to muscle stiffness, spasms, impaired mobility, pain, bladder dysfunction, sleep disturbances, and reduced quality of life. Accurate assessment of spasticity and muscle stiffness is essential, and advanced imaging techniques such as ultrasound elastography have emerged as valuable tools for the in vivo quantification of muscle mechanical properties [10].

The aim of our study was to evaluate the utility of real-time elastography for the objective assessment of muscle status in patients with MS and spasticity, by comparing it with the traditional Ashworth Scale (AS) and complementing these findings with neurophysiological testing (H-reflex, H/M ratio). AS is a widely used clinical tool for assessing muscle tone by grading resistance during passive movement. However, it primarily reflects resistance to passive movement and has limitations in isolating true spasticity [11]. The H-reflex is an electrophysiological analog of the monosynaptic stretch reflex used to evaluate spinal α-motor neuron excitability and the integrity of the Ia afferent pathway. In spasticity assessment, the maximal H-reflex amplitude relative to the maximal direct motor response (Hmax/Mmax ratio) is commonly measured after stimulation of the tibial nerve and recording from the soleus muscle. Patients with spasticity typically demonstrate an increased Hmax/Mmax ratio, reflecting heightened motoneuron excitability and reduced inhibitory control within spinal reflex circuits. Because of this, the H-reflex and H/M ratio are considered useful objective neurophysiological markers that complement clinical measures such as the Ashworth Scale [12].

## 2. Materials and Methods

This single-center observational study was conducted at the Attikon University Hospital, Athens, Greece, between October 2023 and January 2025. The study was approved by the local Ethics Committee (ΕΒΔ 650/18-09-2023), and all participants provided informed consent prior to inclusion. All patients with MS were under the regular care and follow-up of a neurologist at our outpatient clinic and were recruited from this service.

MS patients were diagnosed according to the 2017 McDonald criteria [13]. The diagnosis of MS was established based on a comprehensive diagnostic work-up, including brain and spinal cord magnetic resonance imaging (MRI), cerebrospinal fluid (CSF) analysis and evoked potential studies (visual, somatosensory evoked potentials) when clinically indicated. Inclusion criteria for the MS group were: (1) confirmed diagnosis of MS, (2) presence of lower limb spasticity of any degree, and (3) ability to undergo ultrasound and neurophysiological testing. Exclusion criteria included: (1) history of other neurological or neuromuscular disorders affecting muscle tone, (2) recent botulinum toxin injection or antispastic treatment modification within the last 3 months, (3) orthopedic conditions affecting the lower limbs, and (4) inability to tolerate neurophysiological or ultrasound assessment.

Spasticity in each MS patient was clinically evaluated using the AS. All patients and controls underwent RTE.

RTE was performed by two neurosonologists using a Canon Aplio Ultrasound System (Canon Medical Systems Corporation, 1385 Shimoishigami, Otawara-shi, Tochigi 324-8550, Japan) equipped with an 18-MHz linear probe (Canon Medical Systems, Otawara-shi, Japan). For each site, light repetitive freehand compressions were applied. Consistent and adequate compression was ensured by the system’s real-time quality indicators. Examinations were conducted with patients and healthy controls in the prone position, and the gastrocnemius muscle of both legs was assessed, with medial and lateral muscle bellies examined bilaterally. Each neurosonologist independently evaluated elastographic images of the medial and lateral gastrocnemius muscles on both sides and assigned scores using the Muscle Elastography Multiple Sclerosis Score (MEMSs), a previously described semi-quantitative 5-point ordinal scale ranging from 1 (normal, homogeneous muscle elasticity) to 5 (markedly increased stiffness with heterogeneous elastographic pattern) [10], with higher scores indicating greater muscle stiffness. Inter-observer reliability between the two blinded examiners was subsequently assessed.

To complement the elastographic assessment, all patients and healthy controls underwent neurophysiological measurements performed by an experienced neurophysiologist.Neurophysiological measurements were performed using Nihon Kohden Neuropack MEB-9400 EMG instrument, Nihon Kohden Corp., 1-31-4, Nishiochiai Shinjuku-ku, Tokyo 161-8560, Japan. Specifically, the soleus H-reflex was elicited by stimulation of the posterior tibial nerve in the popliteal fossa, and recordings were obtained from the soleus muscle using surface electrodes (with the active electrode placed medial to the tibia, at approximately half the distance between the stimulation site and the medial malleolus). The stimulus frequency was 0.2 Hz, and stimulus pulses were of long duration (1 ms). Current was stepwise increased in small increments. In low intensity, a small H-wave appeared while the M-wave was absent or minimal. In moderate intensity, H-wave amplitude increased, and M-wave began to appear. In higher intensity, the H-wave reached maximal amplitude and then declined. Concurrently, the M-wave enlarges progressively. The H-reflex with the largest amplitude was selected for analysis. Supramaximal stimulation was used to obtain the maximal M-wave. Latencies were measured to response onset, and amplitudes were measured peak-to-peak. The H-reflex was recorded bilaterally, with the patient lying on a bed, knees slightly flexed, and without facilitation. For statistical analysis, the following measurements were collected: latency of right H-reflex (R.H.LAT), amplitude of right H-reflex (R.H.AMP), latency of left H-reflex (L.H.LAT), amplitude of left H-reflex (L.H.AMP), R.MH (ratio of M maximum to H maximum amplitude on the right), L.MH (ratio of M maximum to H maximum amplitude on the left). All latencies are measured in milliseconds (ms) and all amplitudes in millivolts (mV).

All examiners (the neurophysiologist and the neurosonologists) were blinded to the participants’ diagnosis, i.e., whether they had MS or were healthy controls.

### Statistical Analysis

Statistical analysis was performed using the Statistical Package for Social Science (SPSS Inc.,version 25.0 for Windows; IBM, Armonk, NY, USA). Statistical analyses were performed using parametric or non-parametric methods depending on data distribution. Continuous variables were assessed for normality using the Shapiro–Wilk test. Normally distributed variables are presented as mean ± standard deviation (SD), whereas non-normally distributed variables are expressed as median and interquartile range (IQR).

Baseline characteristics between patients and controls were compared using an independent samples *t*-test for normally distributed variables and a Mann–Whitney U test for non-normally distributed variables. Categorical variables, including sex, were compared using the chi-square test.

Between-group comparisons of neurophysiological and elastography variables were performed using an independent samples *t*-test or Mann–Whitney U test, as appropriate according to data distribution.

Correlation analyses were performed in the patient group only. Given the ordinal nature of the Ashworth Scale and the non-normal distribution of several variables, Spearman’s rank correlation coefficient (ρ) was used to assess associations between the Ashworth score and neurophysiological and elastography variables. Additionally, correlations between neurophysiological parameters (including H-reflex latency and amplitude, and M-wave parameters) and elastography measurements were evaluated using Spearman’s correlation.

To control for multiple comparisons, *p*-values were adjusted using the Benjamini–Hochberg false discovery rate (FDR) method, with adjusted *p*-values < 0.05 considered statistically significant. All statistical tests were two-tailed, and a *p*-value < 0.05 was considered statistically significant.

Inter-rater reliability for elastography measurements was assessed using quadratic weighted Cohen’s kappa (κ), comparing measurements obtained by two independent raters for each muscle group. Agreement was interpreted as poor (<0.20), fair (0.21–0.40), moderate (0.41–0.60), good (0.61–0.80), or excellent (>0.80).

## 3. Results

A total of 26 MS patients and 27 controls were included. Patients were older than controls (median age 48.0 years [IQR 42.0–56.0] vs. 38.5 years [IQR 33.0–65.0], *p* = 0.092), although this difference did not reach statistical significance. Sex distribution also did not differ between groups (50.0% vs. 33.3%, *p* = 0.341). In the MS group, 11 patients had progressive MS, of whom three were classified as primary progressive and eight as secondary progressive, while 15 patients had relapsing-remitting MS.

Between-group comparisons demonstrated significant differences in selected elastography measurements after correction for multiple comparisons. Specifically, patients exhibited higher values for R.GNM.MED.1, R.GCN.LAT.1, L.GCN.MED.1, and L.GCN.LAT.1 compared with controls (all FDR-adjusted *p* < 0.05). No significant differences were observed for H-reflex latency or amplitude measures, M-wave indices, or the remaining elastography variables after FDR correction (Table 1).

**Figure 1:**
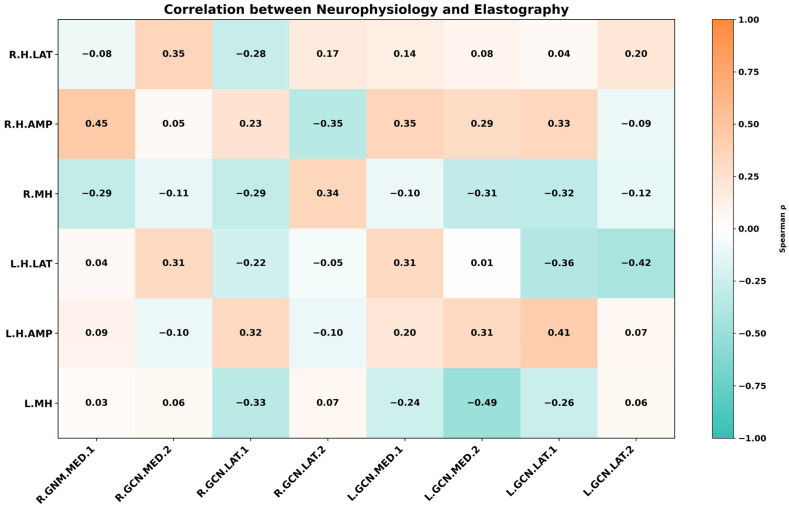
Heatmap showing Spearman correlation coefficients between neurophysiological and elastography variables in patients. Values represent Spearman’s ρ. No correlations remained statistically significant after correction for multiple comparisons. Abbreviations: GCN, gastrocnemius; L, left; R, right; H.LAT, H-reflex latency; H.AMP, H-reflex amplitude; MH, M-wave to H-reflex ratio; MED, medial; LAT, lateral.

In the patient group, the Ashworth score demonstrated a moderate inverse correlation with left gastrocnemius medialis (ρ = −0.56, *p* = 0.016). No other statistically significant correlations between Ashworth score and neurophysiological or elastography variables were identified. Following adjustment for multiple comparisons using the FDR method, this association did not remain statistically significant (Figure 1).

Analysis of the association between neurophysiological parameters and elastography measurements identified correlations between L.MH and L.GCN.MED.2 (ρ = −0.49, *p* = 0.022), and R.H.AMP and R.GNM.MED.1 (ρ = 0.45, *p* = 0.032). No other significant associations were observed. After FDR correction, none of these correlations remained statistically significant.

**Figure 2:**
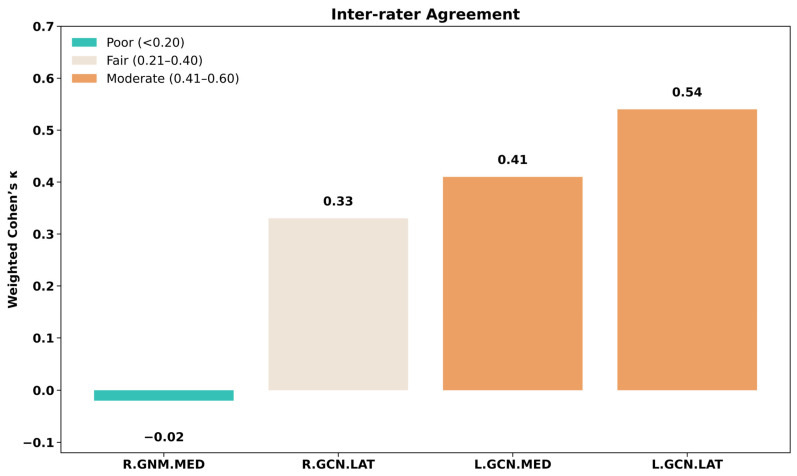
Inter-rater agreement for elastography measurements was assessed using quadratic weighted Cohen’s kappa (κ). Bars represent agreement between two independent raters for each muscle group. Colors indicate level of agreement (poor: <0.20, fair: 0.21–0.40, moderate: 0.41–0.60). Agreement ranged from poor to moderate across measurements. Abbreviations: GCN, gastrocnemius; L, left; R, right; MED, medial; LAT, lateral.

Inter-rater agreement for elastography measurements ranged from poor to moderate (Figure 2). Agreement for R.GNM.MED measurements was poor (κ = −0.02), while R.GCN.LAT measurements demonstrated fair agreement (κ = 0.33). Moderate agreement was observed for L.GCN.MED (κ = 0.41) and L.GCN.LAT (κ = 0.54).

## 4. Discussion

In this single-center study, we explored the potential of RTE for the objective assessment of muscle status in MS patients with spasticity. To the best of our knowledge, this is the first study aiming to validate MEMSs in MS spasticity beyond the original introduction by Illomei et al. [10] and to compare elastography measurements with neurophysiological and clinical findings.

Although spasticity is common in many central nervous system (CNS) disorders and a major source of disability for patients, its measurement and evaluation remain challenging [14]. Methods of spasticity assessment can be broadly classified as clinical or laboratory-based [7]. Clinical evaluation includes physical examination findings (e.g., presence of clonus, triple flexion reflex, other pathological reflexes, passive and active joint range of motion, voluntary muscle strength, contractures, and functional impairment) as well as the use of clinical scales. The most commonly used scale is the Ashworth Scale (AS) [11]. This scale grades muscle tone from 0 (normal) to 4 (severe spasticity) and can be easily applied in a clinical setting without the need for additional equipment. Although the AS is a useful tool, inherent limitations restrict its validity. The main concern is that it quantifies resistance to passive movement rather than isolating spasticity per se [15]; thus, measurements may be influenced by factors unrelated to increased muscle tone, such as intrinsic muscle stiffness and contractures. Furthermore, the AS is limited in its ability to detect changes in muscle tone over time.

For these reasons, instrumented measures have been proposed as more objective and reliable approaches for quantifying spasticity. Neurophysiological methods are commonly used to objectively assess spasticity and treatment effects. Several neurophysiological techniques are described in the literature and can be classified according to the applied stimulus: electrical (H-reflex and F-wave), mechanical (tendon reflex), and passive or active movement paradigms (stretch reflex), while recording muscle action potentials [16]. All of these tests assess components of the phasic stretch reflex but are influenced by different supraspinal pathways. The tendon reflex and stretch reflex are not pure measures of α-motoneuron excitability, as γ-motoneuron activity also contributes to the response. Therefore, these reflexes are considered oligosynaptic rather than monosynaptic, as is assumed for the H-reflex. Due to their oligosynaptic nature, they are considered less standardized and less reproducible compared with the H-reflex [16]. According to the literature, these neurophysiological tests show only moderate to poor correlation with each other as well as with clinical measures (e.g., ROM, AS), and consequently, their validity has been questioned due to limited reliability and sensitivity.

The lack of objective and reliable tests for assessing spasticity has driven the exploration of novel techniques to address this gap [17]. In this context, RTE offers the advantage of complementing conventional ultrasound by adding functional information to anatomical imaging, allowing semi-quantitative evaluation of muscle elasticity in real time [18]. Accordingly, MEMSs were proposed as a promising tool for this purpose [8]. This study included a control group of age- and sex-matched healthy individuals, allowing direct comparison of muscle elasticity between MS patients and healthy subjects. Moreover, to further evaluate its validity, MEMSs’ findings were systematically compared with established clinical measures of spasticity, and all participants underwent H-reflex testing, a widely used neurophysiological marker of spinal α-motoneuron excitability [8]. By integrating RTE-based assessment of gastrocnemius muscle stiffness with H-reflex parameters, our study aimed to provide a comprehensive evaluation encompassing both peripheral mechanical alterations in muscle tissue and central neurophysiological mechanisms contributing to spasticity.

Because the clinical usefulness of a new method depends not only on its ability to detect differences between groups but also on whether it provides results consistent with established assessment tools, evaluation of agreement was a necessary step in validation. Therefore, we assessed the degree of concordance between MEMSs and established measures in (a) the discrimination between healthy controls and MS patients and (b) the grading of spasticity severity.

The analysis demonstrated low concordance between MEMSs and both clinical and neurophysiological assessments across these domains. In particular, several higher MEMSs were not consistently associated with higher clinical spasticity grades. Moreover, neurophysiological tests themselves did not reliably distinguish MS patients from healthy individuals, further highlighting the limited diagnostic performance and heterogeneity of currently available spasticity measures.

Inter-observer reliability [17] for MEMSs scoring was also low, indicating variability in the interpretation of real-time elastography images between examiners. This finding highlights a potential limitation in the use of MEMSs as a standalone diagnostic tool for the assessment of spasticity using ultrasound elastography. Consequently, while MEMSs provide semi-quantitative information on muscle elasticity, their results should be interpreted with caution and ideally complemented by clinical and neurophysiological assessments to ensure a more accurate evaluation of spasticity.

In contrast, neurophysiological assessments did not involve inter-rater reliability, as recordings were obtained by a single examiner and yielded quantitative electrophysiological parameters (e.g., H-reflex latencies and amplitudes) directly derived from the recording system rather than subjective interpretation. Consequently, variability in these measurements is primarily attributable to physiological and technical factors rather than observer disagreement, rendering them more objective, although not necessarily more sensitive for detecting spasticity in this population.

Taken together, apart from clinical evaluation, neither MEMSs nor neurophysiological measures reliably distinguished MS patients from healthy controls nor consistently graded spasticity. This reflects the broader challenge in spasticity assessment, where clinical scales are practical but subjective, and instrumented methods, while more objective, require further validation and specialized expertise. MEMSs provide complementary semi-quantitative information on muscle elasticity but cannot yet be considered a standalone diagnostic tool.

Our study has several limitations. First, the sample size was relatively small, which may limit the generalizability of our findings. Second, while examiners were blinded to participants’ group allocation, complete blinding was not feasible, as spasticity—particularly in patients with higher EDSS scores—cannot be fully concealed during neurophysiological or imaging assessments. These factors may have introduced potential bias and should be considered when interpreting the results.

## 5. Conclusions

Overall, our findings demonstrate a lack of consistent and clinically meaningful association between clinical spasticity severity, neurophysiological measures, and elastography parameters, along with limited inter-rater reliability of MEMSs. This indicates suboptimal reproducibility and limits its validity as a reliable biomarker of spasticity, thereby supporting its use only as a complementary rather than a standalone assessment tool.

## Figures and Tables

**Table 1:** Comparison of neurophysiological and elastography variables between patients and controls.

Variable	Patients	Controls	Test	FDR-Adjusted *p*-Value
R.H.LAT (ms)	31.30 (30.00–32.30)	30.50 (28.20–31.55)	Mann–Whitney U	0.222
R.H.AMP (mV)	0.45 (0.28–1.42)	0.55 (0.38–1.68)	Mann–Whitney U	0.707
R.MH	3.67 (2.17–10.59)	4.62 (1.74–9.88)	Mann–Whitney U	0.915
L.H.LAT (ms)	31.05 (29.07–32.92)	30.20 (29.00–31.70)	Mann–Whitney U	0.574
L.H.AMP (mV)	0.60 (0.18–1.07)	0.80 (0.20–1.40)	Mann–Whitney U	0.707
L.MH	4.22 (2.22–17.00)	4.86 (2.00–25.00)	Mann–Whitney U	0.915
R.GNM.MED.1	2.00 (2.00–3.00)	1.00 (1.00–2.00)	Mann–Whitney U	**0.013**
R.GCN.MED.2	2.00 (1.00–3.00)	2.00 (2.00–3.00)	Mann–Whitney U	0.915
R.GCN.LAT.1	2.00 (2.00–3.00)	1.00 (1.00–2.00)	Mann–Whitney U	**0.007**
R.GCN.LAT.2	3.00 (2.00–3.00)	2.00 (1.00–3.00)	Mann–Whitney U	0.110
L.GCN.MED.1	3.00 (2.00–3.00)	1.00 (1.00–2.00)	Mann–Whitney U	**0.007**
L.GCN.MED.2	3.00 (2.00–4.00)	2.00 (2.00–3.00)	Mann–Whitney U	0.078
L.GCN.LAT.1	2.00 (2.00–3.00)	1.00 (1.00–2.00)	Mann–Whitney U	**0.012**
L.GCN.LAT.2	3.00 (2.00–4.00)	2.00 (2.00–3.00)	Mann–Whitney U	0.198

Abbreviations: GCN, gastrocnemius; L, left; R, right; H.LAT, H-reflex latency; H.AMP, H-reflex amplitude; MH, M-wave to H-reflex ratio; MED, medial; LAT, lateral.

## Data Availability

The datasets used and analyzed during the current study are included in this article. More detailed datasets are available from the corresponding author on reasonable request.

## Abbreviations

The following abbreviations are used in this manuscript:

MS	Multiple Sclerosis
AS	Ashworth Scale
RTE	Real-time ultrasound elastography
MEMSs	Muscle Elastography Multiple Sclerosis Score
FDR	false discovery rate
CNS	Central nervous system
GCN	gastrocnemius
L	left
R	right
MED	medial
LAT	lateral
H.LAT	H-reflex latency
H.AMP	H-reflex amplitude
MH	M-wave to H-reflex ratio

## References

[1] Ophir J., Céspedes I., Ponnekanti H., Yazdi Y., Li X. (1991). Elastography: A quantitative method for imaging the elasticity of biological tissues. Ultrason. Imaging.

[2] Gennisson J.L., Deffieux T., Fink M., Tanter M. (2013). Ultrasound elastography: Principles and techniques. Diagn. Interv. Imaging.

[3] Itoh A., Ueno E., Tohno E., Kamma H., Takahashi H., Shiina T., Yamakawa M., Matsumura T. (2006). Breast disease: Clinical application of US elastography for diagnosis. Radiology.

[4] Pallwein L., Mitterberger M., Struve P., Pinggera G., Horninger W., Bartsch G., Aigner F., Lorenz A., Pedross F., Frauscher F. (2007). Real-time elastography for detecting prostate cancer: Preliminary experience. BJU Int..

[5] Drakonaki E.E., Allen G.M., Wilson D.J. (2009). Real-time ultrasound elastography of the normal Achilles tendon: Reproducibility and pattern description. Clin. Radiol..

[6] Klauser A.S., Faschingbauer R., Jaschke W.R. (2010). Is sonoelastography of value in assessing tendons?. Semin. Musculoskelet. Radiol..

[7] Hugos C.L., Cameron M.H. (2019). Assessment and Measurement of Spasticity in MS: State of the Evidence. Curr. Neurol. Neurosci. Rep..

[8] Nielsen J.B., Crone C., Hultborn H. (2007). The spinal pathophysiology of spasticity—From a basic science point of view. Acta Physiol..

[9] Pandyan A.D., Gregoric M., Barnes M.P., Wood D., Van Wijck F., Burridge J., Hermens H., Johnson G. (2005). Spasticity: Clinical perceptions, neurological realities and meaningful measurement. Disabil. Rehabil..

[10] Illomei G., Spinicci G., Locci E., Marrosu M.G. (2017). Muscle elastography: A new imaging technique for multiple sclerosis spasticity measurement. Neurol. Sci..

[11] Harb A., Margetis K., Kishner S. (2025). Modified Ashworth Scale. StatPearls.

[12] Voerman G.E., Gregoric M., Hermens H.J. (2005). Neurophysiological methods for the assessment of spasticity: The Hoffmann reflex, the tendon reflex, and the stretch reflex. Disabil. Rehabil..

[13] Thompson A.J., Banwell B.L., Barkhof F., Carroll W.M., Coetzee T., Comi G., Correale J., Fazekas F., Filippi M., Freedman M.S. (2018). Diagnosis of multiple sclerosis: 2017 revisions of the McDonald criteria. Lancet Neurol..

[14] Malhotra S., Pandyan A.D., Day C.R., Jones P.W., Hermens H. (2009). Spasticity, an impairment that is poorly defined and poorly measured. Clin. Rehabil..

[15] Bohannon R.W., Smith M.B. (1987). Interrater reliability of a modified Ashworth scale of muscle spasticity. Phys. Ther..

[16] He J., Luo A., Yu J., Qian C., Liu D., Hou M., Ma Y. (2023). Quantitative assessment of spasticity: A narrative review of novel approaches and technologies. Front. Neurol..

[17] Drakonaki E.E., Allen G.M., Wilson D.J. (2012). Ultrasound elastography for musculoskeletal applications. Br. J. Radiol..

[18] Kottner J., Audigé L., Brorson S., Donner A., Gajewski B.J., Hróbjartsson A., Roberts C., Shoukri M., Streiner D.L. (2011). Guidelines for Reporting Reliability and Agreement Studies (GRRAS) were proposed. J. Clin. Epidemiol..

